# Factors Related to Non-participation in the Basque Country Colorectal Cancer Screening Programme

**DOI:** 10.3389/fpubh.2020.604385

**Published:** 2020-12-11

**Authors:** M. Solís-Ibinagagoitia, S. Unanue-Arza, M. Díaz-Seoane, L. Martínez-Indart, A. Lebeña-Maluf, I. Idigoras, I. Bilbao, I. Portillo

**Affiliations:** ^1^BioCruces-Bizkaia Health Research Institute, Barakaldo, Spain; ^2^Department of Nursing I, Faculty of Medicine and Nursing, University of the Basque Country UPV/EHU, Leioa, Spain; ^3^Department of Preventive Medicine and Public Health, University Clinical Hospital of Valladolid, Valladolid, Spain; ^4^Basque Country Colorectal Cancer Screening Programme, Osakidetza, Basque Health Service, Bilbao, Spain

**Keywords:** social inequalities, colorectal cancer, screening programme, risk factors, prevention, non-participation

## Abstract

**Background:** Despite the high participation rates in the Basque Country, colorectal cancer screening programme (Spain), there is still a part of the population that has never participated. Since it is essential to ensure equal access to health services, it is necessary to identify the determinants of health and socio-economic factors related to non-participation in the screening programme.

**Methods:** Cross sectional descriptive study including all invited population in a complete round between 2015 and the first trimester of 2017. Health risk factors available in medical records and their control have been analyzed using univariate and multivariate analyses.

**Results:** 515,388 people were invited at the programme with a 71.9% of fecal immunochemical test participation rate. Factors that increase the risk of non-participation are: being men (OR = 1.10, 95% CI 1.09–1.12); younger than 60 (OR = 1.18, 95% CI 1.17–1.20); smoker (OR = 1.20, 95% CI 1.18–1.22); hypertensive (OR = 1.14, 95% CI 1.12–1.15) and diabetic (OR = 1.40, 95% CI 1.36–1.43); having severe comorbidity (OR = 2.09, 95% CI 2.00–2.19) and very high deprivation (OR = 1.15, 95% CI 1.12–1.17), as well as making <6 appointments to Primary Care in 3 years (OR = 2.39, 95% CI 2.33–2.45). Still, the area under the curve (AUC) indicates that there are more factors related to non-participation.

**Conclusions:** The participation in the Basque Country colorectal cancer-screening Programme is related to some risk factors controlled by Primary Care among others. Therefore, the involvement of these professionals could improve, not only the adherence to the CRC screening, but also other health styles and preventive interventions.

## Background

After 10 years of implementation, the Bowel Cancer Screening Programme (BCSP) of the Basque Country has progressively achieved a significant increase in participation, from 58.1% in 2009 to 72.3% in 2017 surpassing European Guidelines recommendations (65%) (1, 2). Literature suggests that high levels of regular participation are required for screening programmes to be effective (3–6).

The BCSP is based on Primary Health Care (PHC), where most of the preventive and health promotion activities are carried out. A full description of this programme can be found as reported by Portillo et al. (7).

The main results after the first invitation indicate higher colorectal cancer (CRC) detection; however, the incidence has been progressively descending over time according with successive rounds participation. In addition, more than 70% of the cancerous lesions detected by screening were in earlier stages (I and II) than CRC detected in non-participants and survival at 5 years was significantly higher (90.1 vs. 60.5%) (3).

Despite our high participation rate, there are people who having been invited by the programme have never participated, even though all the process is completely free of charge without appointment to get and drop off the screening test. CRC screening participation could be influenced by several factors (socio-economic factors, lifestyles, comorbidities, health preventive actions...). Some of these factors may be related to the determinants of health or to the relationship that the population has with the health system. Therefore, knowledge of determinants of health inequality involved is necessary to address the problem.

Some of the socio-economic factors that could be related to participation have been studied in the literature. Regarding sex, data show consistently lower levels of participation in men than in women (5), even though advanced adenoma and CRC detection rates are higher in men (8–11). Moreover, the benefits of participation increase in men showing a pronounced decrease in mortality (4.3% in men vs. 1.9% in women) (1, 3).

Furthermore, according to the European Code Against Cancer, there are behaviors that help reduce the risk of developing cancer such as not smoking, reducing alcohol consumption, exercising, and eating healthy (12). Despite this, the literature studying the relationship between these factors and non-participation is scarce, as it focuses mainly on socio-economic inequalities (13).

The aim of the present study is to identify socio-demographic and lifestyle factors related to non-participation in the CRC Screening Programme of the Basque Country (Spain).

## Methods

### Study Design and Settings

A cross sectional descriptive study was conducted between May and June 2019. The CRC screening programme of the Basque Country procedures consists of an immunochemical test [fecal occult blood test (FOBT), OC-Sensor®] every 2 years, followed by a colonoscopy under deep sedation in positive cases as confirmatory test (≥20 μg hemoglobin/g feces).

### Study Population

All invited population between 2015 and the first trimester of 2017 was included, covering the target population between 50 and 69 years of age in the Basque Country (a complete round). Persons were totally excluded if they had previously been diagnosed with a CRC, or temporarily excluded if a colonoscopy was performed in the last 5 years. People with unknown addresses were considered invalid invitees. Finally, 515,388 people were invited appropriately.

### Study Variables: Independent Variables

Based on the literature, age, sex, smoking, and obesity (before the invitation) were considered risk factors for development of CRC (14) and diabetes mellitus and arterial hypertension as prevalent chronic conditions. Sex, not gender, was considered due to available data from medical records.

Furthermore, we also included the use of the health services [any type of general practitioner (GP) or nurse consultation apart from those for administrative purposes], participation in preventive activities (influenza vaccine), control of certain risk factors (diabetes, obesity, and arterial hypertension) by PHC following a protocol. Comorbidity and deprivation indexes were calculated using five socio-economic indicators selected from each small area: unemployment, insufficient instruction, insufficient instruction in young people, manual workers, and temporary wage-earners (see Additional File 1 in Supplementary Material) (15, 16). The sample population was also categorized into five groups according to their participation in previous rounds.

The variables were categorized as shown in the Additional File 2 (Supplementary Material).

### Study Variables: Dependent Variables

Non-participation was considered the main outcome in this study. An invited person was considered non-participant if they did not have an FOBT valid result. If the FOBT result was erroneous and no other valid sample had been delivered, it was also considered a non-participant. The database was cleaned eliminating duplicates, leaving us only with the last invitation.

After that, if the result of the FOBT was positive, the adherence of a consequent colonoscopy or another complementary imaging test is required. This variable was measured taking into account that a person with a positive FOBT has subsequently undergone a colonoscopy or conclusive alternative test, so that the quality of the preparation is adequate and the entire colon is observed until cecum. Lesions detected in colonoscopy are coded following the European Guidelines as normal (including hyperplastic polyps), low risk adenoma (LRA), medium risk adenoma (MRA), high-risk adenoma (HRA), other intestinal pathologies, and CRC (2). The most severe result was considered for each participant. Quality indicators and complications on the colonoscopy are described as well.

### Data Sources

Information was obtained from the Basque Country BCSP Database, which has a system of encryption and access in accordance with the current data protection laws, and standardized medical record (Osabide), that belongs to the Basque Health Service (Osakidetza) and permits an effective coordination between PHC and specialized care. Moreover, PHC has some specific facilities included in the medical record to prioritize the detection and control of risk factors and follow-up preventive interventions such as diabetes, hypertension, tobacco consumption, alcohol, and obesity among others (17). All data were systematically anonymized for analysis and subsequent publication.

### Statistical Analysis

The study population was described using frequencies and percentages for categorical variables and means and standard deviations (SDs) for continuous variables. For categorical variables, χ^2^-test was used or Fisher's test when the expected frequencies were <5. Univariate and multivariate logistic regression were conducted to estimate Odds Ratios (ORs) with 95% confidence intervals (CIs), considering statistical significance at the 5% level (*p* ≤ 0.05). Models were systematically adjusted by sex. Discrimination was measured by the area under the receiver operating characteristic curve (AUC).

In addition, each subpopulation with the risk factor (hypertensive, obese, and diabetic) was considered and checked to see if the control of this factor influences non-participation. The same was done with participation in other preventive activities measured by flu vaccination in ≥65-year olds for whom it is recommended.

If a patient had not visited a GP or nurse in the last 3 years, the absence of information in certain variables (tobacco consumption, arterial hypertension, diabetes, and obesity) was considered as missing data and it was excluded from the analysis.

The analysis was carried out using the statistical program SPSS 23.0, IBM (Armonk, New York, USA).

## Results

515,388 people were invited in the study period. The participation rate was 71.9% (74.1% women, 69.4% men), 5.3% were positive and the adherence for colonoscopy was 93.8%. A flow-chart with all details is shown in Additional File 3 (Supplementary Material).

The invited people were 52% women and their mean age was 58.7 (SD = 5.8). The characteristics of the invited population by sex are shown in Table 1. Non-participation levels were significantly higher in men than in women (30.6 vs. 25.9%) as well as advanced adenomas (44 vs. 26.2%) and carcinoma findings (4.6 vs. 3.5%). About half of the population was a regular participant (53.2%). However, a 23.1% of people had never participated (women 22%, men 26.7%).

**Table 1:** Characteristics of the invited population for CRC screening program by sex.

	**Sex**				**Total**		***p***
	**Women**		**Men**				
	***n***	**%**	***n***	**%**	***n***	**%**	
**Age**							<0.001
50–60 years	163,175	60.6	154,567	62.7	317,742	61.7	
61–71 years	105,881	39.4	91,765	37.3	197,646	38.3	
**Type of participant**							<0.001
Regular participant	150,136	55.8	124,216	50.4	274,352	53.2	
Non-participant	59,140	22.0	65,768	26.7	124,908	24.2	
Irregular participant	15,616	5.8	14,024	5.7	29,640	5.8	
Initial participant	26,931	10.0	24,512	10.0	51,443	10.0	
Successive participant	17,233	6.4	17,812	7.2	35,045	6.8	
**Participation**							<0.001
Participants	199,442	74.1	171,037	69.4	370,479	71.9	
Non-participants	69,614	25.9	75,295	30.6	144,909	28.1	
**Findings**							<0.001
Normal/Hyperplasic polyps	3,957	49.2	3,038	29.4	6,995	38.1	
Relevant non-neoplastic pathology	90	1.1	105	1.0	195	1.1	
Low risk adenomas	1,605	20.0	2,172	21.0	3,777	20.6	
Advanced adenomas	2,110	26.2	4,541	44.0	6,651	36.2	
Carcinoma	283	3.5	474	4.6	757	4.1	
**Comorbidity index**							<0.001
Very low	70,876	26.3	68,962	28.0	139,838	27.1	
Low	158,941	59.1	134,964	54.8	293,905	57.1	
Moderate	30,112	11.2	31,610	12.8	61,722	12.0	
Severe	4,850	1.8	7,085	2.9	11,935	2.3	
Missing	4,277	1.6	3,711	1.5	7,988	1.5	
**Deprivation index**							<0.001
Very low	58,628	21.8	50,961	20.7	109,589	21.3	
Low	51,043	19.0	46,729	19.0	97,772	19.0	
Moderate	52,716	19.6	48,535	19.7	101,251	19.6	
High	49,517	18.4	45,724	18.6	95,241	18.5	
Very high	42,894	15.9	40,455	16.4	83,349	16.2	
Missing	14,258	5.3	13,928	5.6	28,186	5.5	
**Tobacco**							<0.001
Smoker	74,195	27.6	94,120	38.2	168,315	32.7	
Non-smoker	175,148	65.1	128,968	52.4	304,116	59.0	
Missing	19,713	7.3	23,244	9.4	42,957	8.3	
**Obesity (BMI ≥ 30)**							<0.001
Obese	27,089	10.1	29,663	12.0	56,752	11.0	
Non-obese	222,221	82.6	193,402	78.6	415,623	80.6	
Missing	19,746	7.3	23,267	9.4	43,013	8.4	
**Arterial hypertension**							<0.001
Hypertensive	62,775	23.3	79,120	32.1	141,895	27.5	
Non-hypertensive	187,651	69.8	145,683	59.1	333,334	64.7	
Missing	18,630	6.9	21,529	8.8	40,159	7.8	
**Diabetes**							<0.001
Diabetic	16,418	6.1	27,922	11.3	44,340	8.6	
Non-diabetic	233,073	86.6	195,423	79.4	428,496	83.1	
Missing	19,565	7.3	22,987	9.3	42,552	8.3	
**Influenza vaccine (≥65 years)**							<0.001
Vaccinated	19,337	33.1	19,080	38.4	38,417	35.5	
Non-vaccinated	39,149	66.9	30,668	61.6	69,817	64.5	
**Primary care visits**							<0.001
≤	63,395	23.6	74,559	30.3	137,954	26.8	
7–15	64,278	23.9	59,852	24.3	124,130	24.1	
16–28	68,966	25.6	56,338	22.9	125,304	24.3	
≥29	72,332	26.9	55,532	22.5	127,864	24.8	
Missing	85	<0.1	51	<0.1	136	<0.1	

*BMI, body mass index*.

In terms of health risk factors of the invited population, we observed that men had a higher proportion of smokers (38.2 vs. 27.6%), obese (12.0 vs. 10.1%), hypertensive (32.1 vs. 23.3%), and diabetics (11.3 vs. 6.1%) than women. However, men use less PHC services than women, in fact, 45.4% of men visit PHC 16 times or more, compared to 52.5% of women. On the contrary, influenza vaccination was superior in men (38.4 vs. 33.1%).

A multivariate analysis was performed including the previously mentioned statistically significant variables in the univariate analysis. First, it was made with all the population and then the disaggregated analysis by sex was repeated. The results of global analysis are shown in Table 2. Regarding the analysis by sex the results showed the same trend as the global analysis, except obesity that shows to be protective in men (OR = 0.96, 95% CI 0.93–0.99, *p* = 0.015) (see Additional File 4 in Supplementary Material).

**Table 2:** Multivariate logistic regression.

**Variables**	**OR**	**95% CI**
**Sex (ref. Women)**		
Men	1.10	1.09–1.12
**Age (ref. 61–71 years)**		
50–60 years	1.18	1.17–1.20
**Comorbidity index (ref. Very low)**		
Low	0.88	0.86–0.89
Moderate	1.21	1.18–1.25
Severe	2.09	2.00–2.19
**Deprivation index (ref. Very low)**		
Very high	1.15	1.12–1.17
High	0.92	0.90–0.94
Moderate	0.86	0.84–0.88
Low	0.86	0.84–0.88
**Tobacco (ref. Non-smoker)**		
Smoker	1.20	1.18–1.22
**Diabetes (ref. Non-diabetic)**		
Diabetic	1.40	1.36–1.43
**Arterial hypertension (ref. Non-hypertensive)**		
Hypertensive	1.14	1.12–1.15
**Primary care visits (ref**. **≥29)**		
≤6	2.39	2.33–2.45
7–15	1.44	1.41–1.47
16–28	1.15	1.12–1.17

*All the variables showed a statistically significant association (p < 0.001)*.

Adjusting for the other variables, men were more likely not to participate in the screening programme (OR = 1.10, 95% CI 1.09–1.12). With regard to age, it was observed that people in the younger group have a higher risk of not participating than those older than 60 years old (OR = 1.18, 95% CI 1.17–1.20). When it comes to lifestyle, smokers are also at greater risk (OR = 1.20, 95% CI 1.18–1.22). Having hypertension (OR = 1.14, 95% CI 1.12–1.15) or diabetes (OR = 1.40, 95% CI 1.36–1.43) means an increase in the probability of not participating.

**Figure 1:**
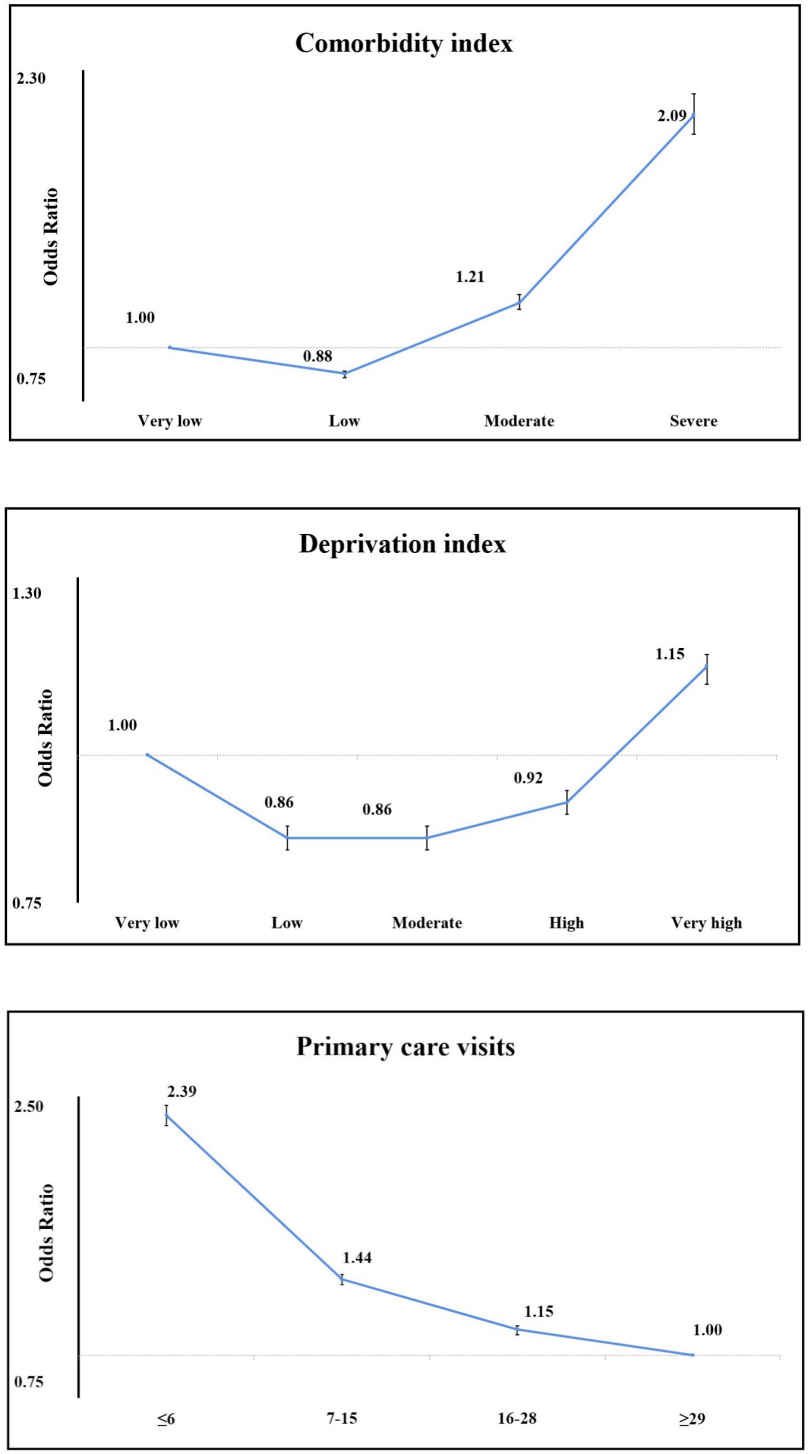
ORs for non-participation in CRC screening by comorbidity, deprivation index, and PHC visits.

Comorbidity and deprivation index as well as PHC visits were classified in more than two categories and are represented in Figure 1. Studying comorbidity index, the risk of non-participation increased as the index descended, and this drop was more pronounced in both moderate to severe. Deprivation index showed the same trend but the differences between categories were slighter. After a slight decrease in the risk of non-participation of those who have a low deprivation with respect to those who have a very low index, the risk increases as the deprivation index increases.

Finally, PHC visits reflected a marked gradient (Figure 1), so that the group of the least number of visits (≤6 visits) had double the risk of not participating (OR = 2.4, 95% CI 2.33–2.45) in comparison to the reference category (≥29).

The area under the curve was 0.611 (95% CI 0.609–0.613, *p* < 0.001).

Another analysis was performed on subpopulations that presented each risk factor or preventive activity studied (Table 3). The results showed that people with inadequate control of these risk factors or non-participation in preventive activities have an increased risk of not participating in the screening.

**Table 3:** Sub-analysis (univariate) of risk factors control.

**Variables**	**OR**	**95% CI**
**Influenza vaccine (ref. Vaccinated)**		
Non-vaccinated	1.88	1.82–1.94
**BMI (ref. Measured at last 2 times/2 years)**		
Non-measured at last 2 times/2 years	1.27	1.21–1.32
**SBP (ref**. **<140 mmHg)**		
>140 mmHg	1.24	1.18–1.32
**DBP (ref**. ** <90 mmHg)**		
>90 mmHg	1.36	1.31–1.42
**HbA1c (ref**. ** <7.6%)**		
>7.6%	1.7	1.61–1.79

*All the variables showed a statistically significant association (p < 0.001)*.

*BMI, body mass index; SBP, systolic blood pressure; DBP, diastolic blood pressure; HbA1c, glycosylated hemoglobin*.

First, the hypertensive with a systolic blood pressure (SBP) higher than 140 mmHg have a 24% higher risk of not participating (OR = 1.24, 95% CI 1.18–1.32) and those with a diastolic blood pressure (DBP) higher than 90 mmHg have a 36% increased risk of not participating (OR = 1.36, 95% CI 1.31–1.42).

For diabetics, having a Hemoglobin A1c (HbA1c) level higher than 7.6% increased the risk of not participating by 70% (OR = 1.7, 95% CI 1.61–1.79).

Among obese people, we observed that not having at least two body mass index (BMI) measurements in the last 2 years increases the risk of non-participation by 27% (OR = 1.27, 95% CI 1.21–1.32). Finally, in subjects over 65 years old who are recommended for influenza vaccination, the risk of not participating was 88% higher in non-vaccinated people (OR = 1.8, 95% CI 1.82–1.94). All the results were statistically significant (*p* < 0.001).

## Discussion

Sex must be considered as one of the main factors that can determine participation in cancer screening programmes. It has been widely studied, and our results agree with a vast majority of studies that have evidenced that participation in CRC screening programmes around the world is greater in women than in men (18–23).

The literature suggests that men may have worse self-care awareness, which could be reflected by being less active in preventive activities or taking care of themselves (24). This could be explained by the fact that traditional models of masculinity have a higher risk of not participating in CRC screening (25–27). In addition, men are at greater risk of developing CRC so they represent a sector of the population to be taken into consideration in screening. In this study we have seen that older men have a lower risk of not participating. According to Moss et al., one explanation could be that screening becomes more acceptable in successive rounds, which reduced the differences in participation between the sexes in their study (28).

On the other hand, both the comorbidity index and the deprivation index are also important factors to consider. Hall et al. (2013) concluded in a qualitative study that having other health problems is a barrier to participating, as participation in screening is not a priority, due to the fact that they do not consider the risk of cancer in the moment they are invited (29). Probably it may be due to their serious health status that they not consider participating (29, 30). However, van Dam et al. reported that worse physical health reduces the possibility of non-participation due to high use of the health system (31). Moreover, in this relation it is necessary to emphasize that the risk of non-participation of those who had a very low index—those with less comorbidity—was slightly higher than those with a low index. Perhaps because the perception of a healthy status may cause individuals to underestimate the risk of becoming ill and consequently they do not take the test, which is also shown in other studies (32).

Deprivation index shows a similar trend to the comorbidity index, thus the groups of very low and very high deprivation index have the highest risk of non-participation. In the Basque Country, people with a high socio-economic status usually have access to and use private health insurance, even with a health system that provides universal coverage. This could mean that many of these people are being screened outside the public system (33) and they may also have the perception of a lower risk of cancer. On the contrary, those with a high level of deprivation could be focused on problems arising from a precarious situation or have personal difficulties to deliver the sample (34–36). In fact, Dawidowicz et al. found a strong association between social deprivation and non-participation (37).

Furthermore, the relationship between a high deprivation index and an unhealthy lifestyle has been demonstrated, which causes a major proportion of individuals with health risk factors (38) like smoking (39, 40), obesity (41, 42), or diabetes (43–45).

Finally, missing values correspond to people who have not visited the health system in the last 3 years before the invitation, so we were not able to register this data. Nevertheless, this exclusion was random, so there is no bias in the analysis and it does not affect the results.

### Strengths

The sample size is the main strength of our study. Literature that relates health risk factors to non-participation in screening is scarce. Our study is novel because we are not aware of any study that analyses the influence of the control of these factors. For this reason, subsequent studies and design interventions are needed in this area.

### Limitations

Besides, the main limitation of this study could be that well-known health risk factors such as alcohol consumption or an unhealthy diet have not been included as they are not properly registered in the medical records. Moreover, isolation and unwanted loneliness is another factor that we have not been able to register and may encourage non-participation. Probably the value of the area under the curve was insufficient because there must be more factors that we have not analyzed in this study that influence non-participation and they should be explored in future. In addition to this, comparing participation in CRC screening with breast and cervix screening programmes has not been possible. With regard to gender, it has not been possible to analyze its relationship with non-participation because both medical records and most of the literature consider only sex although “gender” and “sex” are commonly used equally when they are not really equivalent. In future, it would be interesting to take gender into account when analyzing people's behavior and its effect on their health.

Adherence to colonoscopy has not been addressed in this study because the factors involved could be widely different. Further investigation in this area is needed.

To conclude, it can be said that PHC has an important role in health prevention, promotion, and control of risk factors. Encouraging PHC to take part by actively giving advice for CRC screening can lead to an increase in participation.

## Conclusions

Being men, young, smoker, diabetic, hypertensive, or under-frequented in PHC increases the risk of being a non-participant in the Basque Country's CRC screening programme. Having poor control of health risk factors, or not actively taking part in preventive activities further increases the chances of not participating.

Non-statistically significant sex differences have been observed in factors affecting non-participation, except for obesity. Non-obese men are at higher risk of not participating than those who are obese.

A higher index of deprivation increases the risk of non-participation. However, people with the lowest deprivation also have high non-participation rates. This implies a social inequality that needs to be considered and that probably requires affirmative action measures.

PHC is a basic pillar when it comes to improving the recruitment of people who do not participate in CRC screening. Higher involvement of PHC would be necessary.

## Data Availability Statement

The raw data supporting the conclusions of this article will be made available by the authors, without undue reservation.

## Ethics Statement

Ethical approval for this study was obtained (08/08/2018) from the Research with Drugs Ethics Committee of the Basque Country (CEIm).

## Author Contributions

SU-A, II, IB, and IP were involved in the conception and design of the study. MS-I, LM-I, AL-M, and IP collected the data, MS-I, AL-M, LM-I, and IP performed the analysis. MS-I, SU-A, MD-S, and IP were primarily involved in the drafting of the manuscript. All authors participated in the interpretation of the results of this study. All authors reviewed and approved the final version of the submitted manuscript.

## Conflict of Interest

The authors declare that the research was conducted in the absence of any commercial or financial relationships that could be construed as a potential conflict of interest.

## Abbreviations

AUC: area under the receiver operating characteristic curve
BCSP: Bowel Cancer Screening Programme
BMI: body mass index
CI: Confidence interval
CRC: colorectal cancer
DBP: diastolic blood pressure
FOBT: fecal occult blood test
GP: general practitioner
HbA1c: Hemoglobin A1c
HRA: high-risk adenoma
LRA: low risk adenoma
MRA: medium risk adenoma
OR: Odds Ratio
PHC: Primary Health Care
SBP: systolic blood pressure
SD: Standard deviation.
