# Supplementary material for: Factors Related to Non-participation in the Basque Country Colorectal Cancer Screening Programme
Source: Front Public Health. 2020 Dec 11;8:604385. doi: 10.3389/fpubh.2020.604385 (PMC7760939; doi:10.3389/fpubh.2020.604385)
Supplement: Supplementary file 1 [file Data_Sheet_1.pdf]

Additional File 1

Description of the Comorbidity index and the deprivation index

1. **Comorbidity index**

The Basque Health Service assign a predictive index to each patient, reviewed every year. The main objective of this index is to plan and organize the investment and resources based on the level of self-management of chronicity trends.

The index is the result of dividing the total healthcare cost in the upcoming 12 months by the average cost predicted for the current population. The cost is a proxy of needs, as the higher the index, the higher the future healthcare investment.

First of all, socio-demographic data (age and sex) were extracted; all diagnosis (ICD-9/ICD-10) registered in the medical record (surgeries, procedures) and discharges (MBDS-Minimum Data Set); pharmacological treatments prescribed (ATC – Anatomical Therapeutic Chemical codes) and healthcare cost in the previous 12 months.

Afterwards, all data extracted are recoded by the case-mix adjustment system (Adjusted Clinical Groups- Johns Hopkins ACG System) in the predictable variables introduced in the final model. The procedure to obtain the risk scores is explained extensively by Orueta et al (2013)[1].

The index was categorized for this study into: very low (self-management), low, moderate and severe (high degree of comorbidities).

2. **Deprivation index**

Dominguez-Berjón et al. in 2008[2] constructed a deprivation index by census tract of five cities in Spain. The objective was to achieve an index that described the socio-economic situation, and allowed the identification of those small areas with the most unfavourable situations to analyse mortality. The MEDEA project has been used in more analyses afterwards, including the Colorectal Cancer Screening in the Basque Country.[3]

After a systematic review, the authors defined several socioeconomic indicators and applying a multivariate methodology, the following indicators were included in the index: manual workers, unemployment, casual workers, and total insufficient education and youth. In this way, the deprivation index categorized in quintiles constitutes a summary measure of the socio-economic characteristics of each of the census tracts.

In the present study, based on the 2011 census, the Programme invitees were linked to the quintile they were assigned in the five deprivation categories: very low, low, moderate, high and very high.

1. Orueta JF, Nuño-Solinis R, Mateos M, Vergara I, Grandes G, Esnaola S. Predictive risk modelling in the Spanish population: A cross-sectional study. BMC Health Serv Res. 2013;13:1. DOI: 10.1186/1472-6963-13-269
2. Domínguez-Berjón MF, Borrell C, Cano-Serral G, Esnaola S, Nolasco A, Isabel Pasarín M, et al. Construcción de un índice de privación a partir de datos censales en grandes ciudades españolas (Proyecto MEDEA). Gac Sanit. 2008;22:179–87.
3. Hurtado JL, Bacigalupe A, Calvo M, Esnaola S, Mendizabal N, Portillo I, et al. Social inequalities in a population based colorectal cancer screening programme in the Basque Country. BMC Public Health 2015; 15:1021. DOI: 10.1186/s12889-015-2370-5.
