# Supplementary material for: Factors Related to Non-participation in the Basque Country Colorectal Cancer Screening Programme
Source: Front Public Health. 2020 Dec 11;8:604385. doi: 10.3389/fpubh.2020.604385 (PMC7760939; doi:10.3389/fpubh.2020.604385)
Supplement: Supplementary file 2 [file Data_Sheet_2.pdf]

Additional File 2. Summary of variables

VARIABLES	CATEGORIES
Age	50-60<br>61-71
Sex	Man<br>Woman
Smoking	Smoker: if affirmative information was registered in the medical records during the last four years<br>Non-smoker
Diabetes mellitus	Diabetic: Code 250 (CIE-10) before invitation<br>Non-diabetic<br>*Among diabetics, it was considered controlled if they had HbA1c≤7.6% in the last measure over eight months before invitation.*
Arterial hypertension	Hypertensive: Code 401-404 (CIE-10) before invitation<br>Non-hypertensive.<br>*Among hypertensives, it was considered controlled if they had SBP≤140mmHg or DBP≤90mmHg in the last three measurements over the last two years*
Obesity	Obese: BMI ≥30 in the last two years<br>Non-obese.<br>*Among the obese, it was considered controlled if they had two or more BMI measurements over the last two years*

Use of the health services (visits to the GP and/or nurse)	≤6<br>7-15<br>16-28<br>≥29
Influenza vaccines (only for people ≥65 years old during the last two years)	Vaccinated<br>Non-vaccinated
Comorbidity index [16]	Very low<br>Low<br>Moderate<br>Severe
Deprivation index [15]	Very low<br>Low<br>Moderate<br>High<br>Very high
Type of participant	Regular participant: participation in the last invitation and in the previous one or more<br>Non-participant: never participated<br>Irregular participant: participation in the last invitation, although not in the previous one or the other way round.

Initial participant (PIN): participation in first invitation in the studied period

Successive participant (PIS): initial participation in second or subsequent invitations
