# Supplementary material for: Factors Related to Non-participation in the Basque Country Colorectal Cancer Screening Programme
Source: Front Public Health. 2020 Dec 11;8:604385. doi: 10.3389/fpubh.2020.604385 (PMC7760939; doi:10.3389/fpubh.2020.604385)

## Additional File 3

## Description of the participation and results of the BCSP of the Basque Country between 2015 and 2017 using a flowchart

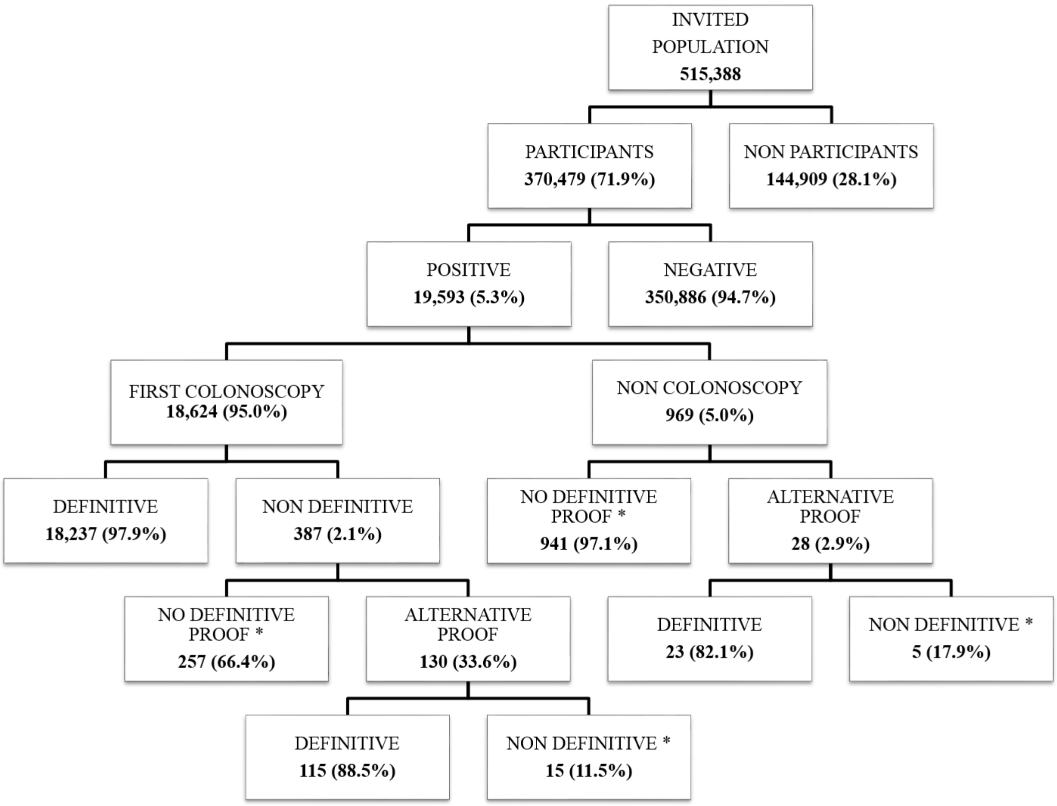

Supplement: Supplementary file 3 [file Data_Sheet_3.pdf]
