# Supplementary material for: Factors Related to Non-participation in the Basque Country Colorectal Cancer Screening Programme
Source: Front Public Health. 2020 Dec 11;8:604385. doi: 10.3389/fpubh.2020.604385 (PMC7760939; doi:10.3389/fpubh.2020.604385)
Supplement: Supplementary file 4 [file Data_Sheet_4.pdf]

Additional File 4.

Multivariate analysis by sex of the factors related to non-participation

1. **Women**

Variables	OR	95% C.I
**Age (ref. 61-71 years)**		
50-60 years	1.13	1.11-1.16
**Comorbidity index (ref. Very low)**		
Low	0.88	0.86-0.90
Moderate	1.20	1.15-1.25
Severe	2.21	2.06-2.36
**Deprivation index (ref. Very low)**		
Very high	1.11	1.08-1.15
High	0.90	0.87-0.93
Moderate	0.84	0.81-0.86
Low	0.85	0.83-0.88
**Tobacco (ref. Non-smoker)**		
Smoker	1.20	1.18-1.23
**Diabetes (ref. Non-diabetic)**		
Diabetic	1.57	1.51-1.63
**Arterial hypertension (ref. Non-hypertensive)**		
Hypertensive	1.24	1.21-1.27
**Primary care visits (ref. ≥29)**		
≤6	2.50	2.41-2.58
7-15	1.44	1.40-1.49
16-28	1.14	1.10-1.17

Note: All the variables showed a statistically significant association ($p<0.001$)

2. **<u>Men</u>**

**Variables**	**OR**	**95% C.I**
**Age (ref. 61-71 years)**		
50-60 years	1.24	1.22-1.27
**Comorbidity index (ref. Very low)**		
Low	0.88	0.86-0.90
Moderate	1.23	1.18-1.27
Severe	2.02	1.91-2.15
**Deprivation index (ref. Very low)**		
Very high	1.17	1.13-1.20
High	0.93	0.90-0.96
Moderate	0.87	0.85-0.90
Low	0.86	0.84-0.89
**Tobacco (ref. Non-smoker)**		
Smoker	1.21	1.19-1.23
**Obesity (ref. Non-obese)**		
Obese	0.96	0.93-0.99
**Diabetes (ref. Non-diabetic)**		
Diabetic	1.32	1.27-1.36
**Arterial hypertension (ref. Non-hypertensive)**		
Hypertensive	1.06	1.03-1.08
**Primary care visits (ref. ≥29)**		
≤6	2.27	2.19-2.35
7-15	1.43	1.39-1.48
16-28	1.17	1.13-1.20

Note: All the variables showed a statistically significant association (all variables $p<0.001$, except “Obesity” $p=0.015$)
